# Phlegmonous Gastritis

**Published:** 1902-02-01

**Authors:** 


### PHLEGMONOUS GASTRITIS.

At the last meeting of the Pathological Society, Dr. Cayley related a curious case of phlegmonous gastritis. The patient was a woman, aged 26, who was admitted to the Middlesex Hospital suffering from pain in the abdomen, tympanites and collapse. Her temperature was 104·6, but no cause for the peritonitis from which she appeared to be suffering could be discovered. She died, and at the necropsy the wall of the stomach was found to be much thickened, and the submucous coat infiltrated with pus. On a bacteriological examination of the exudation being made bacillus coli communis was found in association with streptococcus. It was probable that the infection by streptococcus was primary, and that by bacillus coli a secondary effect. Dr. Cayley, commenting on the case, said that several examples of phlegmonous gastritis had been placed on record. For the production of the condition there were needed, probably, an infective micro-organism and some condition of impaired nutrition of the stomach itself by which its epithelium was rendered less resistant than usual. It was remarkable, he said, in how many cases the patients were of intemperate habits or the attack had followed some excess or error of diet. The condition mentioned had been known to follow injuries and the effects of poisons, and it appeared that a few cases had recovered. It was possible that in the case given the virulent micro-organism had come from some disease of the teeth. The symptoms to be expected in such cases of gastritis would be epigastric pain and tenderness, dilatation of the stomach, vomiting, with possibly the presence of pus in the vomit, high temperature, and the supervention of acute general peritonitis. But these symptoms were by no means always present, and in some cases the disease had run a latent course, its presence being only discovered at the necropsy.

